# Efficacy and tolerance of tacrolimus and pimecrolimus for atopic dermatitis: a meta-analysis

**DOI:** 10.1016/S1674-8301(11)60051-1

**Published:** 2011-11

**Authors:** Zhiqiang Yin, Jiali Xu, Dan Luo

**Affiliations:** a Department of Dermatology;; b Department of Oncology, the First Affiliated Hospital of Nanjing Medical University, Nanjing, Jiangsu 210029, China.

**Keywords:** tacrolimus, pimecrolimus, atopic dermatitis, meta-analysis

## Abstract

Tacrolimus ointment and pimecrolimus cream have proved to be suitable for the treatment of atopic dermatitis. We conducted a meta-analysis of the efficacy, adverse events/withdrawal of tacrolimus versus pimecrolimus in the treatment of atopic dermatitis. According to our meta-analysis, 0.1% tacrolimus was more effective than 1% pimecrolimus in the treatment of adult patients and moderate to very severe pediatric patients, and more 0.1% mild pediatric patients treatal with pimecrolimus withdrew from the trials because of a lack of efficacy or the occurrence of adverse events, compared with mild pediatric patients treated with 0.03% tacrolimus. The combined analyses of tacrolimus with pimecrolimus showed that tacrolimus was more effective than pimecrolimus (week 3: RR=0.67, 95%CI=0.56-0.80; week 6/end of study: RR=0.65, 95%CI=0.57-0.75), and fewer tacrolimus-treated patients withdrew because of a lack of efficacy (RR=0.32, 95CI%=0.19-0.53) or the occurrence of adverse events (RR=0.43, 95%CI=0.24-0.75), compared with pimecrolimus-treated patients. In conclusion, tacrolimus has higher efficacy and better tolerance than pimecrolimus in the treatment of atopic dermatitis.

## INTRODUCTION

Atopic dermatitis (AD) is a chronic, pruritic, and inflammatory skin disorder, which typically commences in early childhood and is characterized by recurrent episodes of relapse and periods of remission. Conventional therapy for AD is based on the use of emollients as maintenance therapy, coupled with short course of topical corticosteroids to treat AD flares. Topical corticosteroids are rapidly effective in treating the acute symptoms of AD, but their use for long-term control of AD are not ideal because of the risk of side effects, such as skin atrophy and suppression of the hypothalamic-pituitary-adrenal axis, which frequently occur in children[1].

Tacrolimus ointment and pimecrolimus cream, two non-steroid topical calcineurin inhibitors, have been used for the treatment of AD over the past 10 years and have proved to be suitable for the treatment of both AD flares and long-term control of AD. Two randomized controlled trials (RCTs)[2],[3] have compared tacrolimus ointment with pimecrolimus cream in the treatment of AD, but the sample size of each study was not large enough to draw definitive conclusions. Thus, we conducted a meta-analysis of RCTs to compare the efficacy, incidences of adverse events and withdrawal (due to a lack of efficacy and due to adverse event) of tacrolimus ointment versus pimecrolimus cream in the treatment of AD.

## MATERIALS AND METHODS

### Literature search and data extraction

We constructed an English-language literature search of PubMed, EMBASE, and the Cochrane Database of Systematic Reviews from January 1998 to March 2011, and considered all RCTs comparing tacrolimus ointment with pimecrolimus cream in the treatment of AD.

Data were extracted from each study by two investigators. Basic information obtained from each eligible study included proportion of patients achieving success of therapy at w 1, 3 and 6/end of study (EOS), and the proportion of patients with any adverse event, withdrawing of patients due to a lack of efficacy, and withdrawing of patients due to adverse event, in each study. Articles were examined to eliminate duplicate reports of the same trial.

The inclusion criteria were as follows: 1) RCTs comparing 0.1% (or 0.03%) tacrolimus with 1% pimecrolimus in the treatment of AD; 2) study medication was applied twice daily to the affected area(s) for up to 6 w or until 1 w after the affected area(s) was entirely cleared, whichever came first; 3) other medicated agents for the treatment of AD were not permitted during the trials. The exclusion criteria were as follows: 1) those randomized patients who did not apply any study medication from the safety (adverse events/withdrawal) analyses; 2) those safety cohort patients who had any major randomization violation from the efficacy analyses were excluded.

### Definition of main outcomes

The definition of “success of therapy” was achieving a score of “clear” or “almost clear” based on the Investigator's Global Atopic Dermatitis Assessment (IGADA). Those patients who used forbidden concomitant medication during the study were included in the final efficacy analyses, but we did not regard the efficacy of those patients as “success of therapy”. In each included study, if all treated areas entirely cleared before the w 6 visit, treatment continued in all areas for 1 additional week, followed by an EOS assessment, but no treatments were continued beyond 6 w.

### Statistical analysis

The methodological quality of the included RCTs was assessed by the Jadad scale. Analysis was performed using the Review Manager version 5.0.25. Statistical heterogeneity assumption among studies was checked by the Chi-square-based *Q*-test. When *I*^2^ was no more than 50%, risk ratio (RR) and 95% confidence intervals (CI) were calculated using the fixed effect model. While significant heterogeneity among the studies was detected, a random effect model was adopted. Estimation of publication bias was made by the funnel plot[4].

## RESULTS

**Fig. 1:**
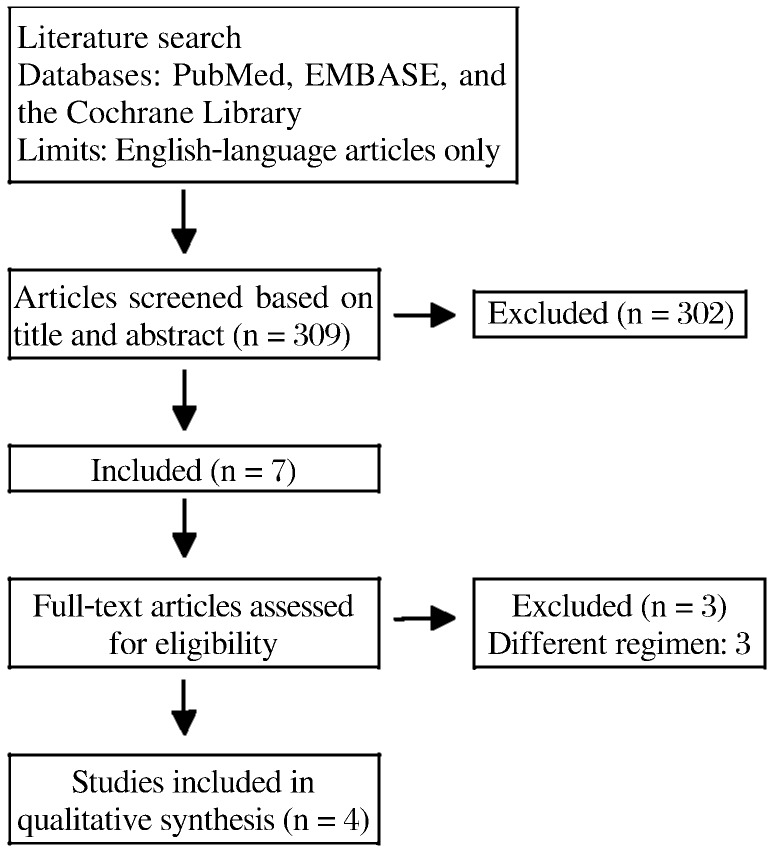
Flow diagram of literature selection.

Four available RCTs[2],[3],[5],[6] with high quality (Jadad score 4) met the inclusion criteria. The study flow diagram is shown in ***Fig. 1***. A total of 1,834 patients were included: 913 received tacrolimus treatment and 921 received pimecrolimus treatment. The cohort for efficacy analyses was composed of 910 tacrolimus patients and 919 pimecrolimus patients (***Table 1***), whereas the cohort for adverse events/withdrawal analyses was comprised of 912 tacrolimus patents and 920 pimecrolimus patients (***Table 2***).

**Fig. 2:**
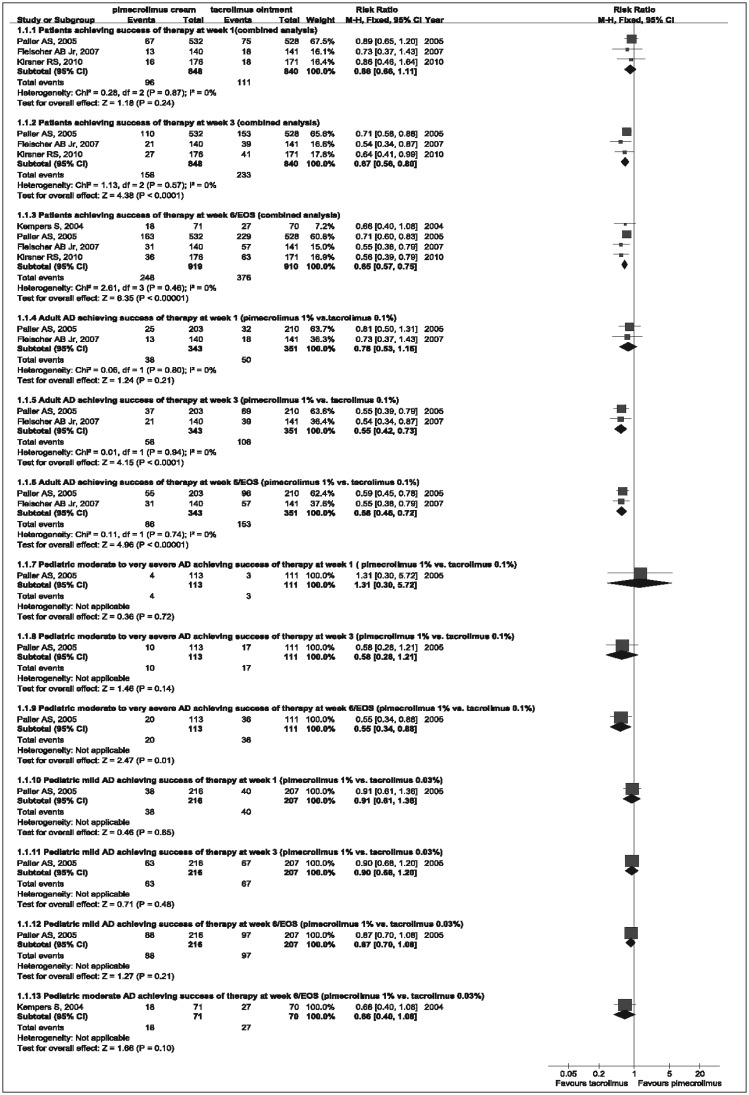
Forest plot with the fixed effect model comparing the efficacy of pimecrolimus cream *vs* tacrolimus ointment at w 1, 3 and 6/EOS. RR and 95%CI for each study and the combined estimate of the efficacy with its CI are plotted on the graph. AD: atopic dermatitis; EOS: end of study.

The evaluable efficacy parameter was “success of therapy” by achieving a score of “clear” or “almost clear”. According to our meta-analysis, tacrolimus 0.1% was more effective than pimecrolimus 1% in the treatment of adult patients (w 3: RR=0.55, 95%CI= 0.42-0.73; w 6/EOS: RR=0.58, 95%CI=0.46-0.72) and moderate to very severe pediatric patients (w 6/EOS: RR=0.55, 95%CI=0.34-0.88), but efficacy analyses had no significant difference between 0.03% tacrolimus and 1% pimecrolimus in the treatment of pediatric mild or moderate pediatric patients. The combined analysis of efficacy in ***Fig. 2*** showed that tacrolimus was more effective than pimecrolimus (w 3: RR=0.67, 95%CI=0.56-0.80; w 6/EOS: RR=0.65, 95%CI=0.57-0.75). The corresponding funnel plot showed a symmetric distribution of studies. Therefore, publication bias did not seem likely.

**Table 1:** Proportion of patients achieving success of therapy at w 1, 3, and 6/EOS in each study

Study	Combined analysis		Adult		Pediatric mild AD		Pediatric moderate AD		Pediatric moderate to very severe AD	
	T	P	0.1%T	1%P	0.03%T	1%P	0.03%T	1%P	0.1%T	1%P
Kempers S, 2004										
Patients(*n*)	70	71	0	0	0	0	70	71	0	0
Success of therapy(*n*)										
Week 6/EOS	27	18	-	-	-	-	27	18	-	-
Paller AS, 2005										
Patients (*n*)	528	532	210	203	207	216	0	0	111	113
Success of therapy(*n*)										
Week 1	75	67	32	25	40	38	-	-	3	4
Week 3	153	110	69	37	67	63	-	-	17	10
Week 6/EOS	229	163	96	55	97	88	-	-	36	20
Fleischer AB, 2007										
Patients (*n*)	141	140	141	140	0	0	0	0	0	0
Success of therapy(*n*)										
Week 1	18	13	18	13	-	-	-	-	-	-
Week 3	39	21	39	21	-	-	-	-	-	-
Week 6/EOS	57	31	57	31	-	-	-	-	-	-
Kirsner RS, 2010										
Patients (*n*)	171	176	61	67	69	71	0	0	41	38
Success of therapy(*n*)										
Week 1	18	16	-	-	-	-	-	-	-	-
Week 3	41	27	-	-	-	-	-	-	-	-
Week 6/EOS	63	36	-	-	-	-	-	-	-	-

EOS: end of study; AD: atopic dermatitis; T: Tacrolimus; P: Pimerolimus.

**Table 2:** Proportion of patients with any adverse event, patients withdrawing due to lack of efficacy, and patient withdrawal due to adverse event in each study

Study	Combined analysis		Adult		Pediatric mild AD		Pediatric moderate AD		Pediatric moderate to very severe AD	
	T	P	0.1%T	1%P	0.03%T	1%P	0.03%T	1%P	0.1%T	1%P
Kempers S, 2004										
Patients(n)	70	71	0	0	0	0	70	71	0	0
Any adverse event	59	61	-	-	-	-	59	61	-	-
Withdrawal										
Due to a lack of efficacy	0	3	-	-	-	-	0	3	-	-
Due to adverse event	1	5	-	-	-	-	1	5	-	-
Paller AS, 2005										
Patients (n)	530	533	210	203	208	217	0	0	112	113
Any adverse event	113	106	67	47	32	36	-	-	14	23
Withdrawal										
Due to a lack Of efficacy	13	35	3	11	4	13	-	-	6	11
Due to adverse event	10	20	6	5	0	10	-	-	4	5
Fleischer AB, 2007										
Patients (n)	141	140	141	140	0	0	0	0	0	0
Any adverse event	42	35	42	35	-	-	-	-	-	-
Withdrawal										
Due to a lack Of efficacy	1	10	1	10	-	-	-	-	-	-
Due to adverse event	3	5	3	5	-	-	-	-	-	-
Kirsner RS, 2010										
Patients (n)	171	176	61	67	69	71	0	0	41	38
Any adverse event	41	45	-	-	-	-	-	-	-	-
Withdrawal										
Due to a lack Of efficacy	4	10	-	-	-	-	-	-	-	-
Due to adverse event	3	10	-	-	-	-	-	-	-	-

AD: atopic dermatitis; T: Tacrolimus; P: Pimecrolimus.

**Fig. 3:**
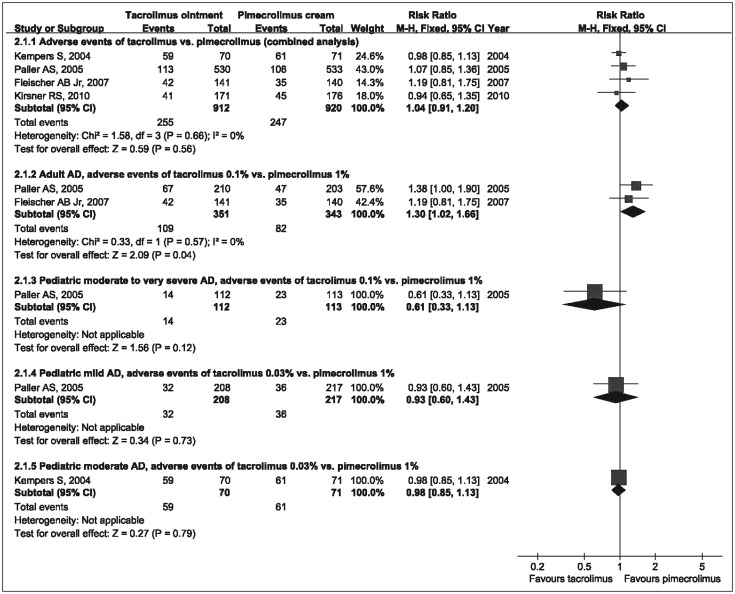
Forest plot with the fixed effect model comparing tacrolimus ointment with pimecrolimus cream in occurrence of adverse events. RR and 95%CI for each study and the combined estimate of adverse events with its CI are plotted on the graph. AD: atopic dermatitis.

According to our meta-analysis of the incidences of adverse events (mostly application site reactions: burning, pruritus, pain and erythema; skin infection; acne; and herpes simplex), 0.1% tacrolimus produced more adverse events than 1% pimecrolimus in adult patients (RR=1.30, 95%CI=1.02-1.66), but there was no significant difference between 0.03% tacrolimus and 1% pimecrolimus in the treatment of mild or moderate pediatric patients, and analysis of the adverse events also showed that 0.1% tacrolimus was similar to 1% pimecrolimus in the treatment of moderate to very severe pediatric patients. The combined analysis of the incidence of adverse events showed that there was no significant difference between tacrolimus and pimecrolimus (***Fig. 3***). The corresponding funnel plot showed a symmetric distribution of studies.

**Fig. 4:**
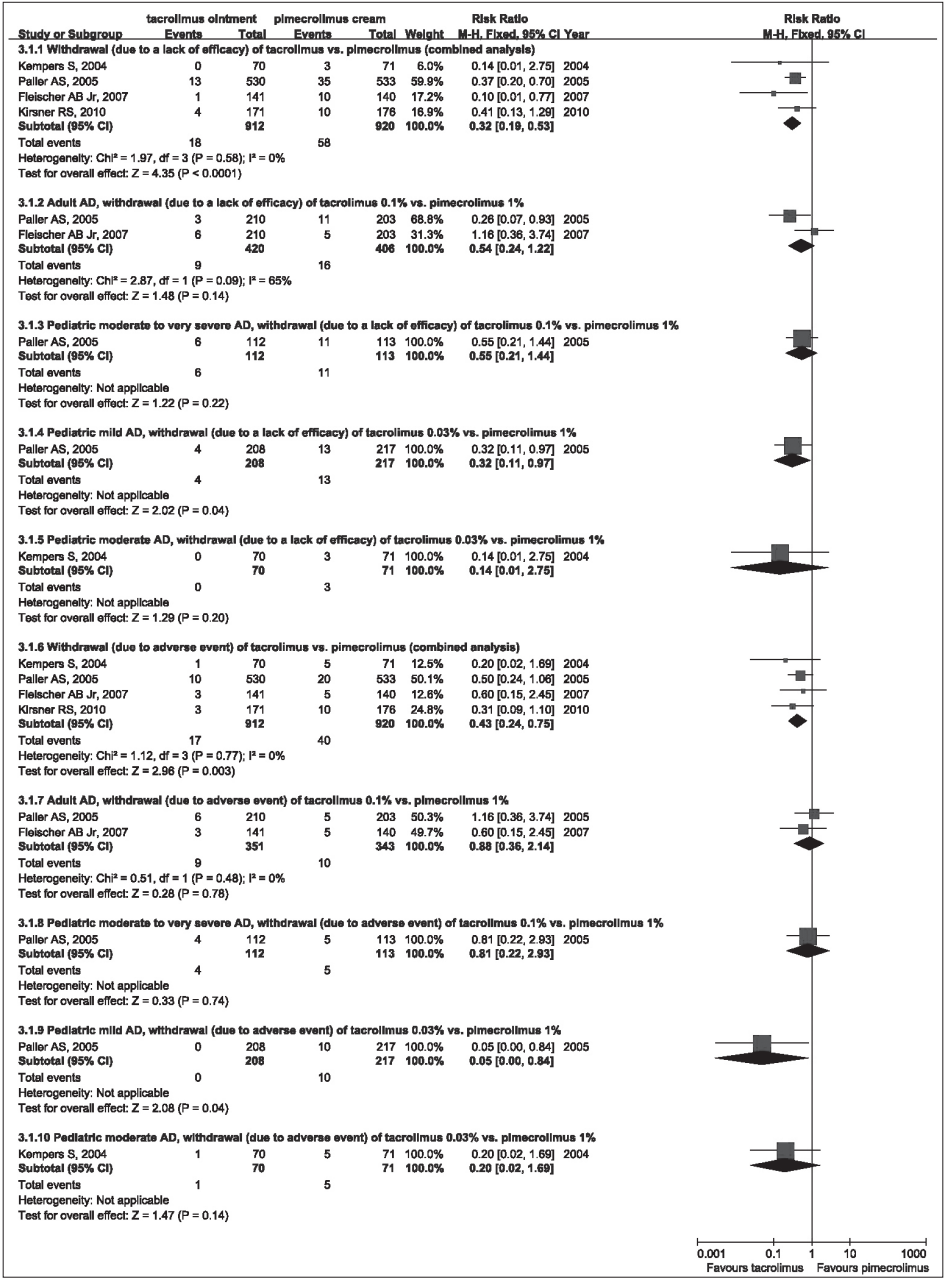
Forest plot with the fixed effect model comparing withdrawal (due to a lack of efficacy and due to adverse event) of tacrolimus ointment vs pimecrolimus cream. RR and 95% CI for each study and the combined estimate of the withdrawal with its CI are plotted on the graph. AD, atopic dermatitis.

Withdrawal analyses showed that fewer mild pediatric patients treated with 0.03% tacrolimus withdrew from the trials because of a lack of efficacy (RR=0.32, 95%CI=0.11-0.97) or adverse events (RR=0.05, 95%CI=0.00-0.84) compared with mild pediatric patients treated with 1% pimecrolimus. There was no significant difference in withdrawal analyses between 0.03% tacrolimus and 1% pimecrolimus in the treatment of moderate pediatric patients, and the withdrawal analyses also showed that 0.1% tacrolimus was similar to 1% pimecrolimus in the treatment of adult patients or moderate to very severe pediatric patients. The combined analyses of withdrawal showed that fewer tacrolimus-treated patients withdrew because of a lack of efficacy (RR=0.32, 95%CI=0.19-0.53) or adverse event (RR=0.43, 95%CI=0.24-0.75), compared with pimecrolimus-treated patients (***Fig. 4***).

## DISCUSSION

This meta-analysis shows that tacrolimus ointment is more effective than pimecrolimus cream at the end of the six-week therapy of AD, and the therapeutic superiority of tacrolimus appears at w 3 and continues through the end of treatment, which especially occurs in the treatment of adult patients with 0.1% tacrolimus *vs* 1% pimecrolimus. Withdrawal due to a lack of efficacy occurs significantly more in pimecrolimus-treated patients than in tacrolimus-treated patients, especially in mild pediatric patients receiving 1% pimecrolimus *vs* 0.03% tacrolimus. There is no significant difference in combined analysis of the incidence of adverse events between tacrolimus and pimecrolimus, but 0.1% tacrolimus produces more adverse events than 1% pimecrolimus in adult patients. Compared with tacrolimus-treated patients, more pimecrolimus-treated patients withdraw because of adverse event, which significantly occurs in the treatment of mild pediatric patients with 1% pimecrolimus *vs* 0.03% tacrolimus, which suggests that pimecrolimus cream has poorer tolerance than tacrolimus ointment.

There are some limitations in our meta-analysis. First, only English-language articles were adopted and the number of the included RCTs was small, only 4. Second, we did not conduct a meta-analysis of cost-effectiveness of tacrolimus ointment *vs* pimecrolimus cream in the treatment of AD, because only one study concerning cost-effectiveness was found, which reported that 0.1% tacrolimus might yield better clinical outcomes and lower costs of care than 1.0% pimecrolimus in adults with AD[7]. Third, our analysis has only compared the short-term efficacy and safety of tacrolimus ointment *vs* pimecrolimus cream in the treatment of AD, because the long-term data comparing tacrolimus ointment with pimecrolimus cream is scarce at present.

In conclusion, tacrolimus ointment is more effective than pimecrolimus cream in the treatment of AD, especially in adult patients and moderate to very severe pediatric patients receiving 0.1% tacrolimus *vs* 1% pimecrolimus, and tacrolimus has higher tolerance than pimecrolimus, which is significant in the treatment of mild pediatric patients with 0.03% tacrolimus *vs* 1% pimecrolimus. In addition, 0.1% tacrolimus produces more adverse events than 1% pimecrolimus in adult patients.

## References

[1] Charman CR, Morris AD, Willams HC (2000). Topical corticosteroid phobia in patents with atopic eczema. Br J Dermatol.

[2] Kempers S, Boguniewicz M, Carter E, Jarratt M, Pariser D, Stewart D (2004). A randomized investigator-blinded study comparing pimecrolimus cream 1% with tacrolimus ointment 0.03% in the treatment of pediatric patients with moderate atopic dermatitis. J Am Acad Dermatol.

[3] Paller AS, Lebwohl M, Fleischer AB, Antaya R, Langley RG, Kirsner RS (2005). Tacrolimus ointment is more effective than pimecrolimus cream with a similar safety profile in the treatment of atopic dermatitis: results from 3 randomized, comparative studies. J Am Acad Dermatol.

[4] Light RJ, Pillemer DB (1984). Summing up: The science of reviewing research.

[5] Fleischer AB, Abramovits W, Breneman D, Jaracz, US/Canada tacrolimus ointment study group (2007). Tacrolimus ointment is more effective than pimecrolimus cream in adult patients with moderate to very severe atopic dermatitis. J Dermatolog Treat.

[6] Kirsner RS, Heffernan MP, Antaya R (2010). Safety and efficacy of tacrolimus ointment versus pimecrolimus cream in the treatment of patients with atopic dermatitis previously treated with corticosteroids. Acta Derm Venereol.

[7] Taneja C, Antaya RJ, Berger A, Marshall TS, Seifeldin R, Oster G (2010). Cost-effectiveness of tacrolimus ointment versus pimecrolimus cream in adults with atopic dermatitis. J Drugs Dermatol.

